#  Possible Foodborne Transmission of Hepatitis E Virus from Domestic Pigs and Wild Boars from Corsica

**DOI:** 10.3201/eid2212.160612

**Published:** 2016-12

**Authors:** Nicole Pavio, Morgane Laval, Oscar Maestrini, François Casabianca, François Charrier, Ferran Jori

**Affiliations:** ANSES (French Agency for Food, Environmental and Occupational Health and Safety);; Maisons-Alfort, France (N. Pavio);; INRA (National Institute for Agricultural Research);; Maisons-Alfort (N. Pavio);; University Paris 12, National Veterinary School, Maisons-Alfort (N. Pavio);; INRA, Corte, France (M. Laval, O. Maestrini, F. Casabianca, F. Charrier);; CIRAD (Agricultural Research for Development);; Montpellier, France (F. Jori);; Botswana University of Agriculture and Natural Resources, Gaborone, Botswana (F. Jori)

**Keywords:** Hepatitis E virus, swine, wild boar, foodborne transmission, Corsica, France, viruses, zoonoses

**To the Editor:** In Western countries, human infection with hepatitis E virus (HEV) is mostly autochthonous and zoonotic through ingestion of contaminated food or direct contact with infected animals and very occasionally is imported from regions to which it is endemic to humans (tropical and subtropical areas) (*1*). Domestic pigs and wild boars are important zoonotic reservoirs of HEV worldwide (*2*).

In continental France, grouped cases of hepatitis E have been described after ingestion of Corsican specialties made with raw pig liver known as ficatelli, traditionally eaten grilled or raw after curing (*3*,*4*). A survey of French food products detected HEV RNA in 30% of ficatelli samples (*5*). A recent nationwide study of blood donors in France showed a high (>60%) HEV seroprevalence in Corsica, suggesting local hyperendemicity (*6*). Estimated prevalences of HEV RNA from wild boars and domestic pigs in Corsica were 2.3% and 8.3%, respectively (F. Jori, unpub. data). We aimed to evaluate, at a molecular level, the role of local wild boars and domestic pigs from Corsica in human infections or food contaminations.

We retrieved partial sequences of HEV open reading frame 2 capsid (*7*) from samples from 8 wild boars hunted during 2009–2013 and from 2 domestic pigs collected at a slaughterhouse in 2013 (F. Jori, unpub. data) and compared them with sequences available in GenBank. This genomic region is used frequently in phylogeny and reflects the diversity of HEV (*8*). After alignment with reference sequences for subtyping (*9*) and their closest sequences, we constructed a phylogenetic tree (Figure). All 10 sequences belonged to HEV genotype 3 and were distributed into 3 distinct clusters.

**Figure:**
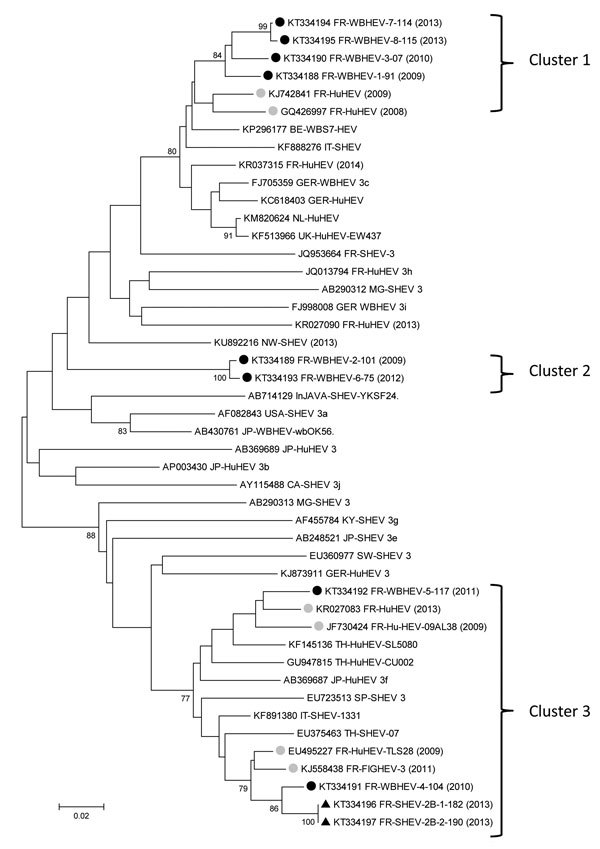
Phylogenetic tree of hepatitis E virus (HEV) sequences identified in samples from wild boars and pigs from Corsica. All 10 HEV sequences (GenBank accession nos. KT334188–KT334197) corresponding to the open reading frame 2 capsid nucleotides 6044–6334 of the reference sequence AF082843, were obtained by Sanger dideoxy sequencing, from wild boars (WB, black circles) or pigs (S, triangles). Sequences were aligned with Muscle (MEGA6, http://www.megasoftware.net) with the 5 closest sequences (retrieved by using BLAST, http://blast.ncbi.nlm.nih.gov/Blast.cgi) and reference sequences (*9*). The closest HEV sequences from France (gray circles; Hu: human, FIG: figatellu) are identified with their GenBank accession number and year of detection. The tree was constructed by using the neighbor-joining method with a bootstrap of 1,000 replicates. Bootstrap values >70% are indicated on respective branches. Three distinct clusters (1–3) are indicated at right. GenBank reference sequences suggested by Smith et al. for genotype subtyping (*9*): 3a, AF082843; 3b, AP003430; 3c, FJ705359; 3e, AB248521; 3f, AB369687; 3g, AF455784; 3h, JQ013794; 3i, FJ998008; 3j, AY115488; AB290312, JQ953664, AB369689, AB290313, EU360977, KJ873911, EU723513. Countries of origin of the sequences used are as follows: CA, Canada; FR, France; GER, Germany; InJAVA, Indonesia; IT, Italy; JP, Japan; KY, Kyrgyzstan; MG, Mongolia; NL, the Netherlands; NW, Norway; SP, Spain; SW, Sweden; TH, Thailand; UK, United Kingdom; USA, United States. Scale bar represents nucleotide substitutions per site.

Cluster 1, subtype 3c, comprised 4 wild boar sequences (FR-HEVWB-1-91, FR-HEVWB-3-07, FR-HEVWB-7-114, FR-HEVWB-8-115) that had 96%–97% nt identity. These sequences were identified during 3 successive hunting seasons (2009, 2010, and 2013) in the same hunting area, suggesting that HEV sequences can be stable, with limited genetic variability, during at least 4 years in a local population of wild boars. These sequences were close to HEV wild boar sequences from Belgium (GenBank accession no. KP296177) and Germany (GenBank accession no. FJ705359; 3c reference sequence). A possible introduction of wild boars from northeast continental France into Corsica during the 1990s could explain such similarity (C. Pietri, pers. comm.). Two human cases reported in southeastern France (GenBank accession nos. GQ426997, KJ742841) in 2008 and 2009 also aggregated within this cluster (94%–95% nt identity), indicating possible zoonotic transmissions from wild boars to humans.

Cluster 2 comprised 2 wild boar sequences (FR-HEVWB-2-101 and FR-HEVWB-6-75) with 99.3% nt similarity, collected in 2009 and 2012 from the same geographic area (Haute Corse, <10 km apart). This cluster is distant from the subtypes assigned by Smith et al. (*9*) and shows <86.5% nt identity with reference sequences (Figure), indicating a possible local and stable evolution in space and time.

Cluster 3, subtype 3f, comprised sequences isolated from wild boars and domestic pigs from Corsica, humans from continental France, and 1 food sample from Corsica. The 2 domestic pig sequences (FR-SHEV-2B-1-182, FR-SHEV-2B-2-190) were 100% identical and shared 97.5% nt identity with a wild boar sequence (FR-HEVWB-4-104), suggesting transmission between domestic and wild pigs. These 2 swine sequences shared 96% nt identity with a sequence amplified in 2011 from a ficatellu sample (FR-HEVFIG-3; GenBank accession no. KJ558438) (*5*) from the same geographic area of Corsica (Haute Corse) and 96% nt identity with an isolate from a patient with acute hepatitis E recorded in France in 2009 (GenBank accession no. JF730424). In addition, the wild boar sequence in this cluster (FR-HEVWB-4-104) shared 96.4% nt identity with the same ficatellu sample and 97.1% nt identity with the same patient in France. This finding suggests that some locally produced ficatelli could be contaminated with HEV from local domestic pigs or wild boars. The human infection also suggests that zoonotic transmission might have occurred through contact with local pig or wild boar reservoirs or through ingestion of contaminated food products. No additional information is available about this human case that might attribute the contamination to 1 of the sources.

Also in cluster 3, another Corsican wild boar sequence (FR-HEVWB-5-117), isolated in 2011, shared 96.2% and 95.7% nt identity with 2 human sequences identified from continental France in 2013 (GenBank accession no. KR027083) and 2009 (GenBank accession no. JF730424 FR-HuHEV-09AL38). This finding again suggests a zoonotic origin for these human cases. Cluster 3 illustrates well a possible path of transmission between wildlife, domestic pigs, food, and human infection and the potential for dissemination of HEV outside Corsica.

Our results provide evidence suggesting a dynamic exchange of HEV between domestic and wild swine reservoirs and possibly resulting in transmission from those reservoirs to humans through ingestion of infected food products. These animal reservoirs are common and abundant (http://www.oncfs.gouv.fr/IMG/file/mammiferes/ongules/ongules_sauvages/TCD/haute_corse_ongules_sauvages_tableau_departemental.pdf; http://draaf.corse.agriculture.gouv.fr/IMG/pdf/Chiffres_cles_Corse-2015_cle825d93.pdf) and represent a sustainable source of HEV exposure in Corsica.

## References

[1] Pavio N, Meng XJ, Renou C. Zoonotic hepatitis E: animal reservoirs and emerging risks. Vet Res. 2010;41:46. 10.1051/vetres/2010018 20359452 PMC2865210

[2] Thiry D, Mauroy A, Pavio N, Purdy MA, Rose N, Thiry E, et al. Hepatitis E virus and related viruses in animals. Transbound Emerg Dis. 2015;n/a; Epub ahead of print. 10.1111/tbed.12351 PMC7169709 25919649

[3] Colson P, Borentain P, Queyriaux B, Kaba M, Moal V, Gallian P, et al. Pig liver sausage as a source of hepatitis E virus transmission to humans. J Infect Dis. 2010;202:825–34. 10.1086/655898 20695796

[4] Renou C, Roque-Afonso AM, Pavio N. Foodborne transmission of hepatitis E virus from raw pork liver sausage, France. [Erratum in: Emerg Infect Dis. 2015;21:384. ]. Emerg Infect Dis. 2014;20:1945–7. 10.3201/eid2011.140791 25340356 PMC4214313

[5] Pavio N, Merbah T, Thébault A. Frequent hepatitis E virus contamination in food containing raw pork liver, France. Emerg Infect Dis. 2014;20:1925–7. 10.3201/eid2011.140891 25340373 PMC4214317

[6] Mansuy JM, Gallian P, Dimeglio C, Saune K, Arnaud C, Pelletier B, et al. A nationwide survey of hepatitis E viral infection in French blood donors. Hepatology. 2016;63:1145–54. 10.1002/hep.28436 27008201

[7] Rose N, Lunazzi A, Dorenlor V, Merbah T, Eono F, Eloit M, et al. High prevalence of hepatitis E virus in French domestic pigs. Comp Immunol Microbiol Infect Dis. 2011;34:419–27. 10.1016/j.cimid.2011.07.003 21872929

[8] Lu L, Li C, Hagedorn CH. Phylogenetic analysis of global hepatitis E virus sequences: genetic diversity, subtypes and zoonosis. Rev Med Virol. 2006;16:5–36. 10.1002/rmv.482 16175650

[9] Smith DB, Simmonds P, Izopet J, Oliveira-Filho EF, Ulrich RG, Johne R, et al. Proposed reference sequences for hepatitis E virus subtypes. J Gen Virol. 2016;97:537–42. 10.1099/jgv.0.000393 26743685 PMC5588893

